# Supplementary material for: Reduction of αSYN Pathology in a Mouse Model of PD Using a Brain-Penetrating Bispecific Antibody
Source: Pharmaceutics. 2022 Jul 5;14(7):1412. doi: 10.3390/pharmaceutics14071412 (PMC9318263; doi:10.3390/pharmaceutics14071412)
Supplement: Supplementary file 1 [file pharmaceutics-14-01412-s001.zip › pharmaceutics-1765412-supplementary.pdf]

SUPPLEMENTARY MATERIAL

Materials and methods

The selectivity of RmAbSynO2-scFv8D3 and RmAbSynO2 to αSYN monomers, oligomers and fibrils was analyzed with an inhibition ELISA. High binding 96-well half-area plates (Corning Inc, Corning, NY, USA) were coated with αSYN monomers diluted in PBS overnight at 4°C and blocked with 1% bovine serum albumin for 1 h. RmAbSynO2-scFv8D3 and RmAbSynO2 (100 ng/mL) were incubated for 2 h with serially diluted αSYN monomers, HNE-induced oligomers and fibrils, starting at 500 nM, 140 nM and 140 nM respectively. Next, pre-incubated antibodies were added to the plates for 15 min at 900 rpm followed by detection with HRP-conjugated anti-mouse-IgG-F(ab')2 (Jackson ImmunoResearch Laboratories, West Grove, PA, USA) and K blue aqueous TMB substrate (Neogen Corp.) and read with a spectrophotometer at 450 nm. All incubations and dilutions were made in PBS with 0.1% BSA, 0.05% Tween, and 0.15% Proclin) and performed in room temperature. IC50, the concentrations of either αSYN monomers, HNE-induced oligomers or fibrils needed to quench half of the ELISA signal was used as an estimate of the antibody's affinity for the investigated antigen. αSYN preparations were made as previously described [1].

Evaluation of binding affinities of RmAbSynO2-scFv8D3 and RmAbSynO2 to HNE-induced αSYN oligomers and mTfR after radiolabeling was performed as previously described [1]. Binding affinity to TfR has been previously investigated for a bispecific antibody with the same scFv8D3-format and determined to be approximately 0.2 nM [2].

Results

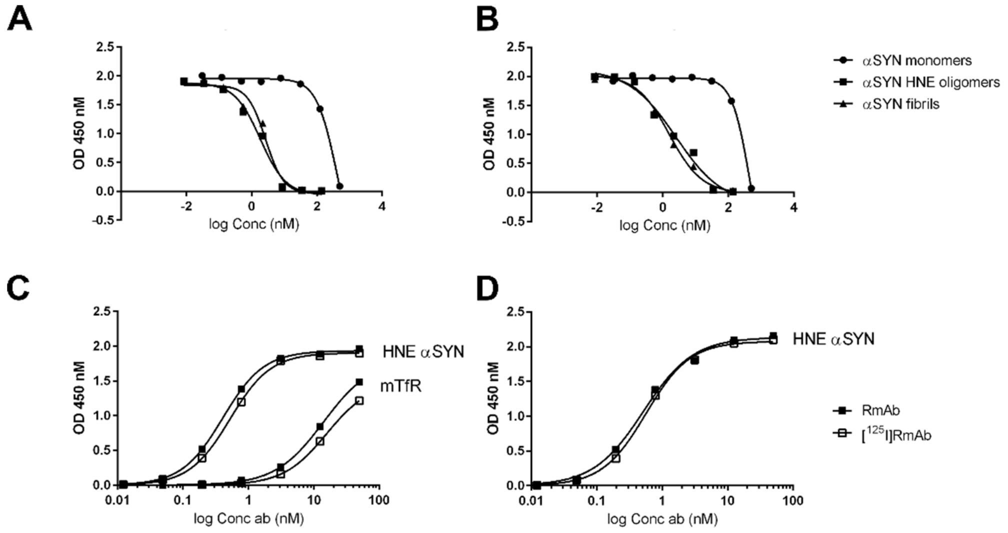

Figure S1. A) – High selectivity for αSYN oligomers and fibrils over monomers displayed by conformation selective antibodies RmAbSynO2-scFv8D3 B) RmAbSynO2. The IC50 for RmAbSynO2-scFv8D3 was determined to be 412±1.30 nM for monomers, 1.85±0.089 nM for oligomers and 2.68±0.10 nM for fibrils and for RmAbSynO2 453±2.3 nM for monomers, 2.53±0.26 nM for HNE oligomers and 1.58±0.085 nM for fibrils. C) Unchanged binding affinities to HNE-induced αSYN oligomers after radiolabeling of RmAbSynO2-scFv8D3 and E) RmAbSynO2, whereas the affinity to mTfR was somewhat lowered. Values are presented as means±SD.

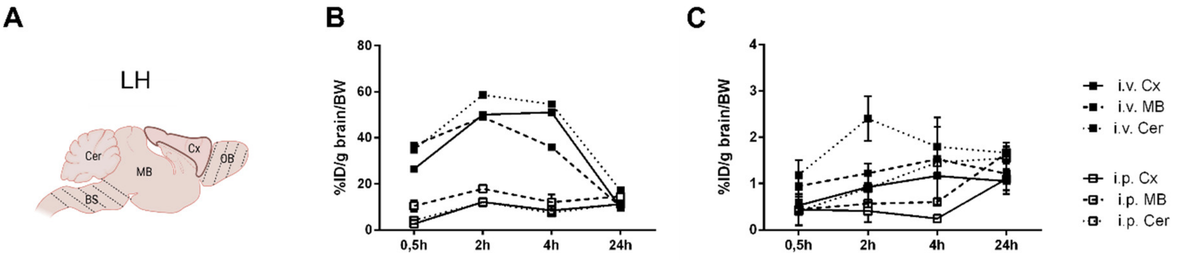

Figure S2. A) Subdissection scheme of left brain hemispheres for examination of regional brain uptake. B) Similar brain uptake between cortex (cx), midbrain (MB) and cerebellum (Cer) for the separate administration routes for RmAbSynO2-scFv8D3 and C) RmAbSynO2-scFv8D3.

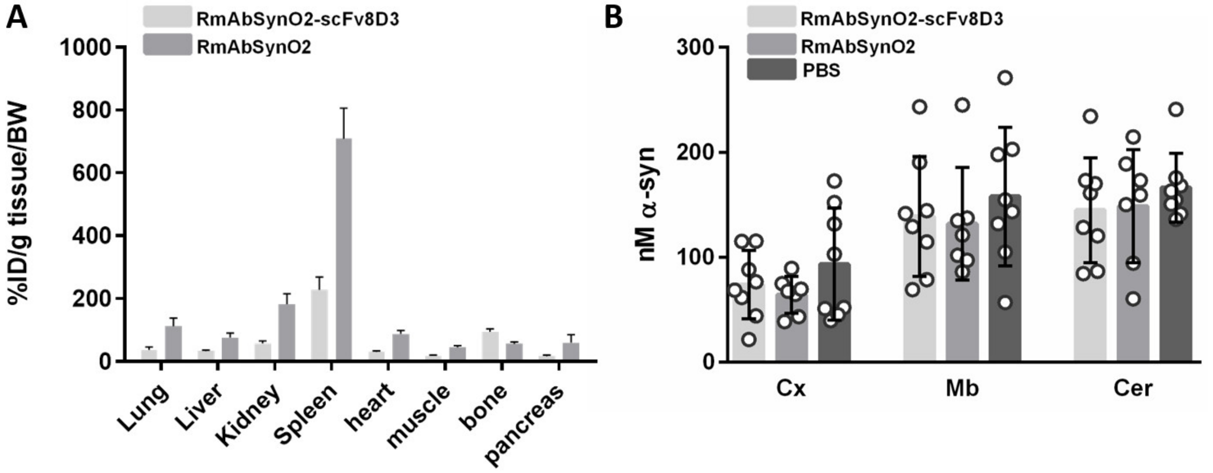

Figure S3. A) Antibody distribution to peripheral organs following three injections (10 mg/kg body weight) of radiolabeled RmAbSynO2-scFv8D3 or RmAbSynO2. B) ELISA quantification of insoluble αSYN levels in formic acid (FA) fraction from cortex (Cx), midbrain (Mb) and cerebellum (Cer), following treatment with RmAbSynO2-scFv8D3 or RmAbSynO2 in comparison with PBS. No differences in αSYN levels were observed between the different treatment groups in comparison with controls. All values

presented as means ± SD, with one-way analysis of variance followed by Sidak's multiple comparison test.

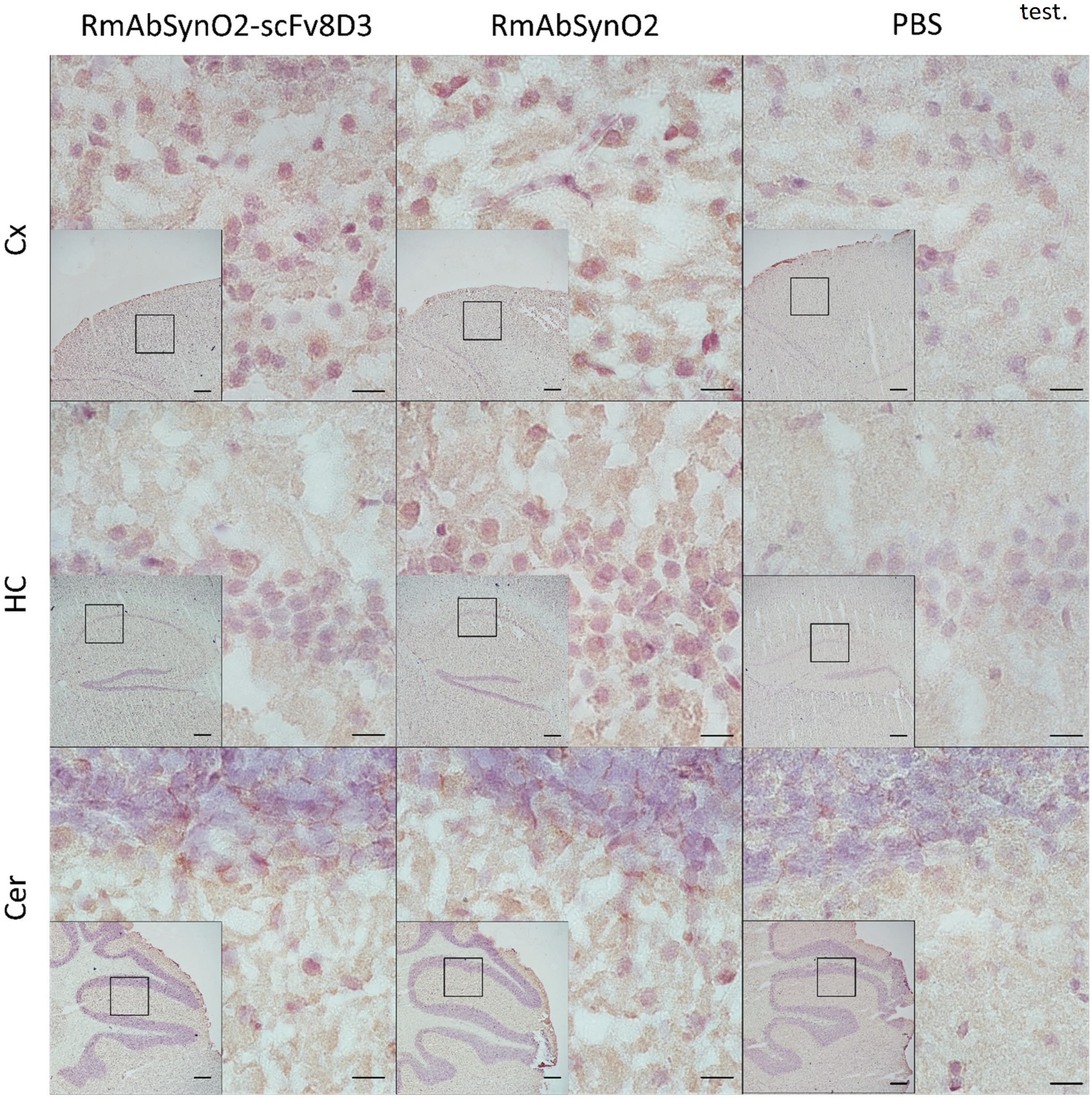

Figure S4. Representative images of 20 µm cryosections stained for TREM2 in Cx, hippocampus (HC) and Cer at 60X magnification, with 4X magnifications embedded with squares representing the magnified area, showing increased immunoreacitvity on sections from mice treated with both antibody formats. Scale bars: 200 µm in embedded images, 10 µm in magnified images.

1. Roshanbin, S., et al., *In vivo imaging of alpha-synuclein with antibody-based PET.* Neuropharmacology, 2022. **208**: p. 108985.
2. Gustavsson, T., et al., *SPECT imaging of distribution and retention of a brain-penetrating bispecific amyloid-β antibody in a mouse model of Alzheimer's disease.* Transl Neurodegener, 2020. **9**(1): p. 37.
